# Change in Urinary Inflammatory Biomarkers and Psychological Health with Gut Microbiome Modulation after Six Months of a Lifestyle Modification Program in Children

**DOI:** 10.3390/nu15194243

**Published:** 2023-10-01

**Authors:** Md Saimul Islam, Shyanne Page-Hefley, Anne P. Hernandez, Luke Whelchel, Chiquito Crasto, Whitney Viator, Treyce Money, Babafela Awosile, Noel Howard, Tetyana L. Vasylyeva

**Affiliations:** 1 Texas Tech University Health Sciences Center, Department of Pediatrics, Amarillo, TX 79121, USA; isl30324@ttuhsc.edu (M.S.I.); lukewhelchel@gmail.com (L.W.); noel.howard@ttuhsc.edu (N.H.); 2 Private Psychologist, 101 SE 18th Ave, Amarillo, TX 79102, USA; 3 Center for Biotechnology and Genomics, Texas Tech University, Canton & Main Experimental Sciences Building, Room 101, Lubbock, TX 79409, USA; chiquito.crasto@ttu.edu; 4 Center for Biotechnology and Genomics, Texas Tech University School of Veterinary Medicine, Amarillo, TX 79106, USA; babafela.awosile@ttu.edu

**Keywords:** adolescents, obesity, inflammation, microbiome, gut–brain axis

## Abstract

**Background:** Obesity is a metabolic disorder that negatively impacts the quality of life. Long-term methods such as exercise and low-fat diets can help regulate this health issue, but 93.3 million Americans continue to struggle. Our research investigates if lifestyle changes can affect urinary inflammation markers and psychological aspects through the modification of gut microbiome composition. **Methods:** Our study included 16 healthy controls with normal BMI as a comparison group and 22 overweight/obese (OW/OB) adolescents. We collected demographic, clinical, psychological, stool, and urine sample data at enrollment and six months after implementing lifestyle modifications. Bacterial genomic data and inflammatory markers in these samples were analyzed. **Results:** The lifestyle interventions were associated with decreased inflammation and enhanced mental health among overweight teens. We observed differences in bacterial community compositions between healthy participants and those who underwent treatment, including exercise and dietary habit adjustments, although there was no significant change in bacterial species richness. Mental health correlated with gut microbiota compositions without any demographic influences. The research also uncovered connections between inflammatory markers, psychological factors, and gut microbiota phyla through carbohydrate metabolism alterations. **Conclusion**: Our findings demonstrate that lifestyle modifications are associated with improved mental health and a reduction in inflammation in overweight adolescents by adjusting the gut microbiota composition.

## 1. Introduction

Obesity has become a significant public health issue. The CDC reported that an alarming 93.3 million people in the United States are obese [1]. It was estimated that 1 out of 5 adolescents struggles with obesity. Obesity’s etiopathogenesis involves genetic, neuroendocrine, metabolic, psychological, environmental, and socio-cultural elements. In recent years, studies have demonstrated the effects of long-term clinical interventions of regular physical activity and low-fat diet consumption on obesity management [2].

Obesity is associated with various severe comorbidities such as diabetes mellitus, hypertension, coronary artery disease, orthopedic issues, impaired quality of life, and negative impacts on self-esteem [3]. Recent evidence suggests that obesity deregulates glucose homeostasis, adipogenesis, thermogenesis, and chronic inflammation by disrupting cellular signaling pathways [4]. A significant cause of this cellular deregulation lies in alterations in gut microbiome composition [5].

The gut microbiome constitutes a complex ecosystem of microorganisms that maintain a symbiotic relationship within their host organism. An imbalance or dysbiosis within this intricate system has been linked to inflammatory disorders [6]. Studies have consistently shown that obese individuals display altered gut microbiota composition compared to their healthy counterparts. The resulting imbalance gives rise to proinflammatory cytokines and neuroactive molecules derived from microbes, subsequently disrupting cytokine levels and hormone balances [7]. Overweight (OW)/obese (OB) individuals often exhibit variations in gut microbial abundance and increased plasma levels of proinflammatory cytokines such as tumor necrosis factor-alpha (TNF-α) and interleukin-6 (IL-6) [8,9].

Additionally, OW and OB individuals have elevated levels of endothelin-1 (ET-1), a marker of endothelial dysfunction linked to hypertension, chronic kidney disease, and stroke [10,11]. The gut microbiome also modifies neuro-inflammatory processes during childhood and adolescence [12]. Studies have shown that enteric microbiota can directly impact the gut–brain axis through neuroendocrine and metabolic pathways while interacting with intestinal cells [13]. 

Despite these advancements, many questions still need to be answered. Our study aimed to explore (i) whether differences exist in non-invasive (urinary) inflammatory markers, psychological parameters, and gut microbiomes among healthy adolescents compared to those who are OW or OB and (ii) investigate whether lifestyle changes can influence urinary inflammatory markers and psychological factors via modulation of gut microbiome composition over a six-month period.

## 2. Materials and Methods

### 2.1. Study Design

The 22 OW/OB (intervention) and 16 healthy (comparison) adolescents were recruited in the study at enrollment. Only 12 OW/OB children were able sustained in a six-month lifestyle modification program. The demographic, clinical, psychological, stool, and urine sample data were collected at enrollment for healthy and OW/OB patients and six months after implementing lifestyle modifications for OW/OB participants. After measurement of inflammatory markers and 16 s bacterial sequencing, correlational analysis was conducted between the species of the gut microbiome and different parameters.

### 2.2. Setting

OW/OB patients were recruited from the outpatient pediatric clinic at the Texas Tech University Health Sciences Center in Amarillo (TTUHSC), TX. Healthy participants were referred by primary care physicians from different health centers around Amarillo. The Institutional Review Board of TTUHSC approved the study. The study subjects and their parents/guardians provided informed assent/consent prior to study participation. Exclusion criteria included males and females younger than eight and older than 18 and participants and/or parent/guardians(s) who did not consent and/or assent to the study.

### 2.3. Participants

Our study used the BMI scale to define healthy, OW, and OB children as per the Centers for Disease Control and Prevention (CDC) [14]. Healthy was defined as having a BMI between the 5th and 85th percentile (*n* = 16) (n who were not on any medications and had no significant medical conditions. Whereas OW (*n* = 9) BMI was between 85th to less than 95th percentile and OB (*n* = 13), BMI ≥ 95th percentile) adolescent males and females aged 8–18 years old were enrolled in the present study. At the beginning of the study, OW and OB participants’ history, demographics, physical exam (blood pressure, height, weight, waist circumference, waist-to-hip ratio, skinfold thickness), and sleep/dietary habits were obtained. Lipid profiles (total cholesterol, LDL, HDL, and triglycerides) and HbA1c were assessed in OW/OB adolescents at enrollment. Furthermore, stool samples were collected from OW/OB adolescents at enrollment. Stool samples were also collected after employing a 6-month lifestyle modification program, which we designated as OW/OB post-intervention. First-morning urine samples were collected at enrollment, 3-months (termed as OW/OB 3), and 6-months. Urine samples were evaluated for inflammatory markers and markers (TNF-α, IL-6) of endothelial dysfunction (ET-1). Healthy adolescents also provided a one-time stool sample at the time of enrollment. 

#### 2.3.1. Psychological Parameters Collection

The Pediatric Symptom Checklist (PSC-Y) Questionnaire was used to assess cognitive, emotional, and behavioral problems in adolescents between the ages of 11 and 18. During enrollment, healthy, OW, and OB adolescents were asked to complete the PSC-Y questionnaire to identify potential mental health problems. The survey evaluates four psychosocial aspects: Possible internalization (depression/anxiety), externalization (conduct disorders, oppositional defiant disorder), attention issues (ADHD), suicidality, and other uncategorized behaviors such as isolation, fatigue, physical complaints, lack of emotional expression, and teacher conflicts. Questions are answered with never, sometimes, or always, scored as 0, 1, and 2. A total score is calculated, with >30 considered positive [15]. Those admitting to suicidal thoughts or attempts were deemed positive regardless of other scores and receive immediate intervention. Unanswered questions were not scored. Participants with positive scores were referred to their doctor for further mental health assessment.

#### 2.3.2. Lifestyle Modification Coaching

As part of our intervention, we implemented a program to improve children’s activity and nutrition by teaching adolescents and caregivers about healthy lifestyles. This 6-lesson interactive course covered energy balance, calories, and exercise. Face-to-face education occurred at enrollment, three and six months later, with monthly phone check-ins for motivation. The initiative was designed as a comprehensive, multi-component approach to address child and adolescent overweight and obesity in TTUHSC pediatric clinics and the Texas Panhandle. Drawing from NIH’s “We Can” program principles. The curriculum focused on better food options, increased activity, and reduced screen time.

Both English and Spanish materials were provided during the training to encourage vital behavior changes in both children and parents working as a team. Each 1 h session included breaks for stretching and movement, with details found in Supplementary 1.

### 2.4. DNA Extraction from Stools and Sequencing

Stool samples were collected from subjects within 24 h prior to a clinical visit. Stool samples were stored at −20 °C until DNA extraction was performed. The samples were processed for DNA extraction using the QIAamp^®^ Fast DNA Stool Mini Kit (Cat No. 51604, Qiagen, Hilden, Germany) according to the manufacturer’s protocol. Each sample’s DNA was amplified by PCR with a bacterial universal primer set, 341F (5′-CCTACGGGNGGCWGCAG-3′) and 805R (5′-GACTACHVGGGTATCTAATCC-3′) against the V3–V4 regions of bacterial 16S rRNA genes. The libraries were then sequenced on a MiSeq sequencer (Illumina Inc., San Diego, CA, USA) using a 600-cycle v3 sequencing kit at the Center for Biotechnology and Genomics, TTU, Lubbock, TX, USA.

### 2.5. Biomarker Assays

ELISA kits were used to assay biomarkers in the urine of healthy, OW/OB and post-intervening OW/OB adolescents. ELISA PicoKine™ kits (BosterBio, Pleasanton, CA, USA) were used to detect tumor necrosis factor-α (TNFα) (Cat No. SKU: EK0525), interleukin-6 (IL-6) (Cat No. SKU: EK0410), and endothelin-1 (ET-1) (Cat No. SKU: EK0945) as per manufacturer protocol. Here, 100 ul of standard (# 1—1000 pg/mL, #2—500 pg/mL, #3—250 pg/mL, #4—125 pg/mL, #5—62.5 pg/mL, #6—31.25 pg/mL, #7—15.625 pg/m) and blank control (sample diluent, i.e., 0 pg/mL) were used in triplicate for the generation of the standard curve. We averaged the triplicate readings for each standard, sample, and control, followed by subtracting the average blank control OD reading. Finally, we generated the standard curve and derived the y = mx + c equation to measure the sample’s protein concentration. If data were outside of the detection limit, they were managed by nonparametric methods [16].

### 2.6. Bioinformatic Analysis Pipeline

The raw data was downloaded and processed using the BLC2FASTQ software developed by Illumina [https://illumina.com, (accesses on 5 June 2023)]. This software converts the raw data from the NGS Microbiome experiment into readable format. Then, raw, readable data are used to generate taxonomic information up to the level of an operational taxonomic unit (OTU) for every bacterial species identified from the experiments using Array-Star 17 software (Version 17) and the PERL (Practical Extraction and Report Language) program. 

After that, OTU data were imported and managed using Microsoft Excel 365 for Windows (2021, Microsoft Corp., Redmond, WA, USA). Then, relative abundance and alpha diversity indices and beta diversity of control, OW/OB at enrollment, and OW/OB after the lifestyle modification were analyzed using R software using different R packages (v4.2.2). A functional profile and differential abundance analysis of the microbiome were performed by NAMCO Microbiome Explorer v1.1 [17]. The built-in PICRUSt2 pipeline and option [18] of NAMCO Microbiome Explorer were used to predict each sample’s functional Kyoto Encyclopedia of Genes and Genomes (KEGG) orthology profile from the relative abundance of OTUs. The detailed analysis process has been described in Supplementary Material 1. 

### 2.7. Statistical Analysis

Biomarker values were expressed as means ± SEM, medians, and interquartile ranges. GraphPad Prism 7.01 (La Jolla, CA, USA) was used for data analysis of various parameters by paired or unpaired *t*-tests and a two-way ANOVA test. Excel Data Analysis Toolpack and GraphPad Prism 8.03 (GraphPad Software, Inc., La Jolla, CA, USA) were used for the statistical analysis of various parameters via descriptive statistics, paired or unpaired *t*-tests, calculation of odds ratios, or one-way ANOVA. *p* < 0.05 was considered to indicate a statistically significant difference. 

## 3. Results

### 3.1. Participants

Healthy (*n* = 16), OW (*n* = 9), and OB (*n* = 13) adolescent males and females aged 8–18 years old were enrolled. Of the 22 OW/OB patients who agreed to participate in the program evaluation, 10 were boys and 12 were girls. Additionally, subject adherence to the program decreased from 22 adolescents with OB/OW at enrollment to 12 participants over three months through 6 months. 

### 3.2. Characteristics 

Only two OW/OB participants were born preterm, and all had appropriate gestational weights at birth. Additionally, four of the participant’s mothers had elevated blood pressure during the pregnancy, while none had gestational diabetes. Assessment of family members revealed that all but one participant had at least one close family member who was obese. In 64% of the participants, the mother was either overweight or obese, and 27% of participants had an obese father. Social characteristics and anthropometric measurements of OW/OB participants are presented in Table 1.

We noted statistically significant differences in weight, BMI, and waist-to-hip ratio between healthy and OW/OB adolescents (Table S1). While adhering to the lifestyle modification program, only 33.3% showed a decrease in weight at six months. However, 68% of participants demonstrated decreased waist-to-hip ratios at six months. No statistically significant changes in BMI z-score were noted after six months.

### 3.3. Measurable Change in Four Gut Microbiome Phyla and Eleven Genera among Prevalent Phyla in OW/OB Adolescents after Employing Lifestyle Modification Program

**Figure 1:**
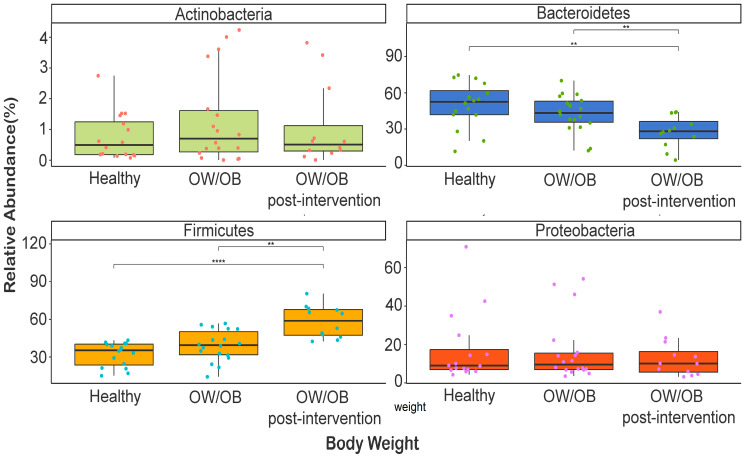
Comparison of relative abundance of dominant phyla between healthy weight, OW/OB, and post-intervention OW/OB participants. The Mann–Whitney statistical comparison between the groups was adjusted for multiple comparisons using Bonferroni correction. ** *p* < 0.01, **** *p* < 0.0001.

In the study’s participants’ gut microbiota, 11 phyla, 22 classes, 39 orders, 64 families, and 72 genera were identified. Body weight influenced the prevalence of Firmicutes (32–58%), Bacteroidetes (27–50%), and Proteobacteria (13–17%) (Figure S1). Healthy individuals had higher Bacteroidetes levels than OW/OB post-intervention participants (*p* = 0.005), whch was also found when comparing overweight versus post-intervention participants (*p* = 0.01) (Figure 1). However, post-intervention participants had higher Firmicutes levels than healthy or overweight individuals (*p* < 0.0001 and *p* = 0.002) (Figure 1). 

**Figure 2:**
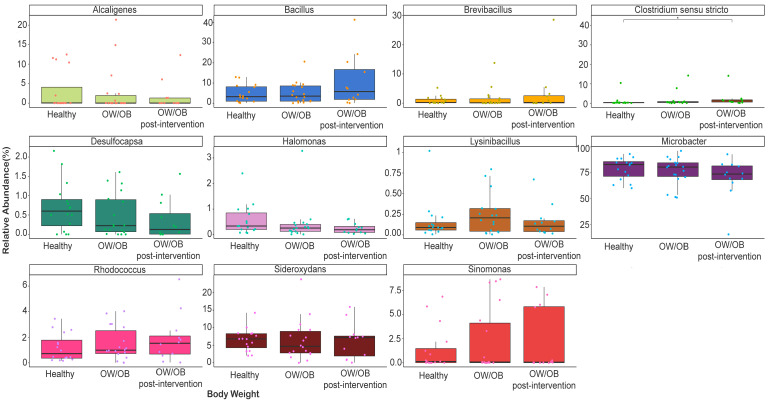
Comparison of relative abundance of dominant genera between healthy weight, OW/OB, and post-intervention OW/OB participants. The Mann–Whitney statistical comparison between the groups was adjusted for multiple comparisons using Bonferroni correction. * *p* = 0.039.

At the genus level, Microbacter spp. (70–78%), Bacillus pp. (4.6–10.6%), Sideroxydans (6.23–6.49%), and Alcaligenes (1.75–2.99%) dominated (Figure S2 and Figure 2). Clostridium senso stricto was higher in post-intervention participants relative to healthy ones (*p* = 0.039) and those with higher ET1 and internalized problems (*p* = 0.033 and *p* = 0.032), and higher in overweight participants compared to healthy participants with frequent internalization issues (*p* = 0.001) (Figure 2). Although it is not significant, the lysinbacillus, Arthrobacter, and micrococcus genera belong to the Firmicutes, and Actinobacteria phylum showed a response to lifestyle modification intervention (Figure 2 and Figure S3). 

### 3.4. Alpha, Beta Diversity Indices Differential Abundance of Microbiomes among the Study Participants

**Figure 3:**
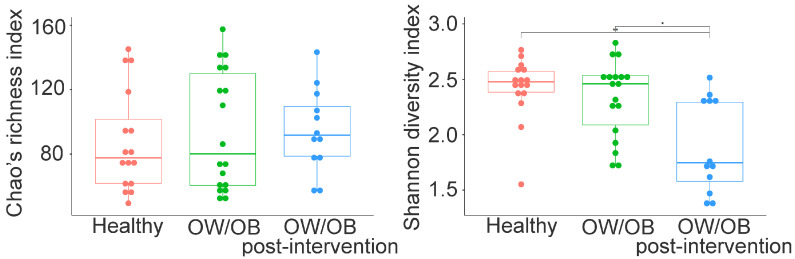
Chao and Shannon diversity indices in gut microbiota between the healthy weight, pre-intervening OW/OB and post-intervening OW/OB participants. Chao index did not differ between the groups, while the Shannon index is statistically different between healthy and treated OW/OB and between OW/OB and treated OW/OB participants. The Mann–Whitney statistical comparison between the groups was adjusted for multiple comparisons using Bonferroni correction. * *p* = 0.002, ** *p* = 0.012.

No significant differences were found in Chao’s species richness among healthy, pre-intervention, and post-intervention OW/OB participants (Figure 3, *p* > 0.05). However, a significant decrease in the Shannon diversity index was observed in treated groups (Figure 3, *p* = 0.002 and *p* = 0.012). Beta diversity analysis using Bray–Curtis dissimilarity and nonmetric multidimensional scaling showed distinct bacterial community compositions among groups (PERMANOVA, *p* < 0.0001) (Figure S4). Significant differences were detected between healthy and post-intervention (*p* = 0.005) and pre-intervention and post-intervention OW/OB participants (*p* = 0.012). 

SIMPER analyses revealed two bacterial genera (Faecalibacterium hattorii and Mangrovibacterium marinum) as driving microbial differential abundance among groups (*p* < 0.01). Faecalibacterium hattorii was significantly higher in post-intervention OW/OB participants compared to others (*p* < 0.0001 and *p* = 0.002), while Mangrovibacterium marinum showed higher levels in healthy weight and pre-intervention OW/OB participants compared to post-intervention OW/OB participants (*p* < 0.01).

### 3.5. OW/OB Adolescents Exhibit Increased Levels of Urinary TNF-α, IL-6, and ET-1 Compared to Healthy Controls

**Figure 4:**
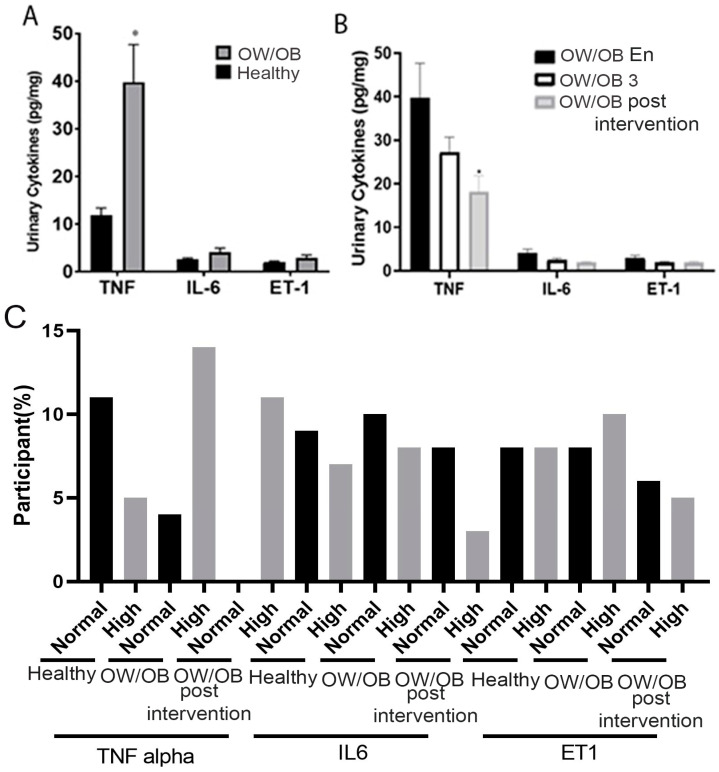
Urinary TNF-α, IL-6, ET-1 levels in OW/OB, OW/OB post-intervention, adolescents, and healthy controls: (**A**) Comparative Urinary cytokine levels between OW/OB and healthy adolescents. (**B**) Comparative urinary cytokine levels of OW/OB adolescents during different time points. (**C**) Frequency of participants who had physiologically normal and high cytokines. OW/OB 3, represents lifestyle modification employed on OW/OB for three months. Normal and high defined based on physiological value. OW/OB En: at enrollment, OW/OB 3: post-intervention over three months, OW/OB post-intervention: over six months. Data represented as mean ± SEM, *p*-value * > 0.05. Unpaired *t*-test.

Urinary levels of TNF-α, IL-6, and ET-1 of OW/OB and healthy adolescents at their enrollment visit are presented in Figure 4. We noted that OW/OB adolescents exhibited significantly higher TNF-α levels than their healthy counterparts. Furthermore, although not statistically significant, we noted an upward trend in urinary IL-6 and ET-1 levels of OW/OB adolescents (1.5 and 1.45-fold increase, respectively) compared to healthy controls (Figure 4). Three and six months after the initial enrollment visit, OW/OB adolescents provided a urine sample, which was processed for TNF-α, IL-6, and ET-1 presence. We noted no statistical differences in the presence of TNF-α, IL-6, and ET-1 at the 3-month follow-up. However, at the 6-month follow-up, we noted a statistically significant decrease in urinary TNF-α levels (2.20-fold decrease) in OW/OB adolescents. Moreover, we observed a downward trend (2.12 and 1.55-fold decrease, respectively) in the levels of urinary IL-6 and ET-1, suggesting that lifestyle and dietary changes may have contributed to the observed decreases (Figure 4B).

Based on the physiological concentration of cytokines, a high percentage of participants showed an increased TNF level compared to healthy ones, which was reduced over the six-month lifestyle modification program (Figure 4C). Our cohort has also observed similar expression patterns of IL-6 and ET 1.

### 3.6. OW/OB Adolescents’ Psychological Behaviors Show Marginal Improvement through Lifestyle Modification Programs

**Figure 5:**
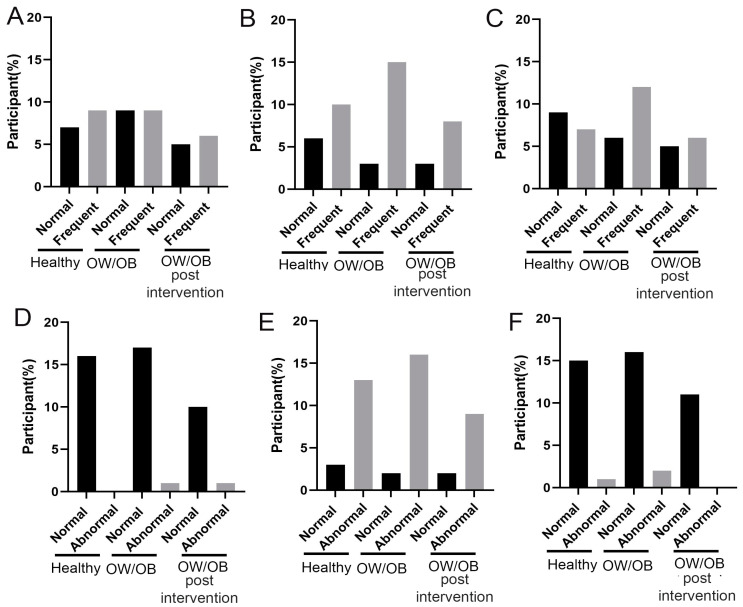
Distribution of psychological parameters score among healthy, OW/OB and after employed six-month lifestyle modification program (OW/OB post-intervention). (**A**) Internalization problem, (**B**) attention problem, (**C**) externalization problem, (**D**) suicidal tendency, (**E**) non-categorical, and (**F**) total score. The percentage of participants indicates that a proportion of participated population in a particular study group has a normal and high cytokine level.

In the majority of OW/OB adolescents, psychological issues were more prevalent compared to their healthy counterparts at the time of enrollment, as assessed by standard psychological measures (Figure 5). Nevertheless, their mental health improved significantly after six months of lifestyle modifications. Among the different psychological factors, internalization, attention, externalization, and non-categorical issues demonstrated a notable rise in the OW/OB group compared to the healthy individuals at enrollment (Figure 5A–C,E,F). However, there was no observed alteration regarding suicidal tendencies throughout this period.

### 3.7. Significant Correlations between Inflammatory and Psychological Indexes across Various Phyla in OW/OB Participants

We examined the link between dominant phyla abundance and participants’ lifestyle, cytokine levels, and mental health (Table 2). We found a higher abundance of Firmicutes in post-intervention OW/OB individuals than in healthy and OW/OB participants (*p* = 0.037 and *p* = 0.022). This trend was also observed with post-intervention OW/OB participants who consumed fruits and vegetables regularly (*p* < 0.01). Higher Firmicutes levels were found in post-intervention OW/OB participants who did not eat outside the home (*p* < 0.03).

Higher Bacteroidetes levels were noticed in OW/OB participants with elevated TNF-alpha (*p* = 0.009) compared to post-intervening OW/OB ones. In comparison, greater Firmicutes levels were seen in post-intervening OW/OB participants with increased TNF-alpha and normal IL-6 (*p* ≤ 0.01). Healthy individuals had higher Bacteroidetes levels than post-intervening OW/OB participants with raised ET1 levels (*p* = 0.019), but a higher Firmicutes abundance was common among the post-intervening group regardless of ET1 levels (*p* < 0.01).

Elevated Bacteroidetes levels were noted among healthy weight and pre-intervening OW/OB adolescents compared to those with abnormal non-categorical negative suicidality and problem-focused tendencies (*p* < 0.02). Conversely, post-intervention OW/OB participants showed higher Firmicutes rates in similar conditions (*p* < 0.04). However, there was no connection between dominant phyla, genera, and participant characteristics.

Lastly, there was no correlation between Chao’s species richness, Shannon diversity index, or lifestyle or clinical attributes. Notwithstanding this point, a significantly higher Shannon diversity index was observed in healthy weight participants and OW/OB participants compared to their post-intervention counterparts with elevated TNF-alpha (median difference = 0.8, *p* = 0.005) or ET1 levels (median difference = 0.89, *p* = 0.019, 0.72, *p* = 0.015, respectively).

### 3.8. Functional Pathway Abundance Differences between Post-Intervening OW/OB Participants Compared to OW/OB and Healthy Participants

**Figure 6:**
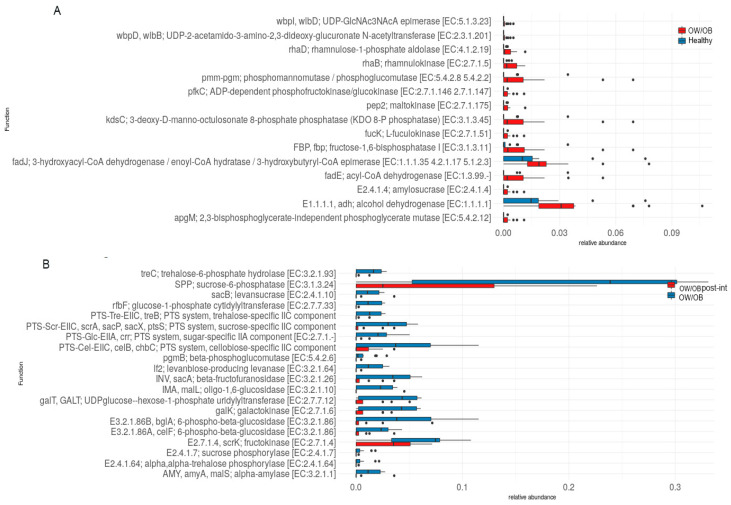
Box plot showing the (**A**) differential relative abundance of carbohydrate metabolism-related KOs between OW/OB (red color) and healthy weight participants (other/blue color). (**B**) Carbohydrate metabolism-related KOs between OW/OB post-intervention (red color) and OW/OB participants (other/blue color).

In the study, most KEGG orthologues (KOs) among participants were related to metabolic pathways and classified into various subcategories. There was a notable difference of 570 KOs between OW/OB and healthy individuals without multiple test corrections; however, none remained significant after using the Benjamini–Hochberg method. Among these, OW/OB participants had a higher abundance of specific metabolic pathways than healthy individuals (Figure 6A) and their post-intervention counterparts (Figure 6B).

Comparing functional profiles between post-intervention OW/OB and healthy participants revealed 1582 significantly different KOs after adjustments. Of these, 433 KOs were more abundant in post-intervention OW/OB participants (Figure S5A), related to carbohydrate metabolism and other metabolic pathways. When comparing treated to pre-intervention OW/OB individuals, 1049 KOs showed significant differences without multiple testing corrections but none after applying the Benjamini–Hochberg method (Figure S5B). Among them, 29 KOs were more abundant in post-intervention OW/OB participants, which was linked to the biosynthesis of co-factors, amino acids, and other metabolic pathways, but not carbohydrate or sugar metabolism.

## 4. Discussion

The relationship between weight, gut microbiome imbalance, and inflammation in overweight and obese adults has been widely researched [19]. However, there is limited data on gut microbiome alterations and non-invasive inflammation markers in obese adolescents undergoing lifestyle modification programs. Introducing lifestyle changes in a family context can be difficult, requiring concerted efforts and consistent reinforcement to establish a routine. Our study found that most participants and their families embraced dietary suggestions, but the six-month period did not yield significant weight reduction.

The complexity and diversity of gut microbiota, crucial for a healthy gut–brain and gut–urinary axis, is highlighted by the presence of various phyla, classes, orders, families, and genera. Interestingly, the post-intervention OW/OB group showed increased Firmicutes abundance but decreased Bacteroide abundance compared to healthy and OW/OB individuals. This finding implies that an imbalance in the ratio of these two microbes contributes to obesity, as reported in other studies [20]. Although the phylum was reduced even after lifestyle modification, it showed a significant association between cytokines and psychological parameters. Additionally, a certain number of genera showed a response to the intervention. The discrepancy might be due to the short period of program implementation. Additionally, a notable decrease in the Shannon diversity index from healthy teens to post-intervention OW/OB participants revealed distinct bacterial community compositions among the groups. A similar decline in diversity (Shannon index) has been observed in an Egyptian adult population [21].

Our study also discovered that OW/OB adolescents had elevated urinary TNF-α, IL-6, and ET-1 levels compared to their healthy counterparts. Although no statistical variances were observed at the 3-month follow-up, the 6-month follow-up indicated a substantial decrease in urinary TNF-α levels alongside downward trends for urinary IL-6 and ET-1 levels, emphasizing the positive impacts of the lifestyle program. These results imply that lifestyle and dietary modifications may have played a role in the observed decreases. The finding is supported by Dod HS et al., who reported significant improvements in IL-6 blood levels following a lifestyle change program involving diet, exercise, and stress management strategies for 12 weeks [22]. A Polish prospective research study also found increased inflammatory cytokine expression in OB individuals [23]. Given the positive outcomes observed after six months of continuous lifestyle intervention, future research should investigate the optimal duration for long-lasting effects.

In addition to these points, psychological studies indicate that OW children display abnormal psychological behaviors compared to their healthier peers. Nevertheless, through adopting lifestyle changes, adolescent participants in this program showed an enhancement in mental health, particularly in areas such as internalization, attention, externalization, and non-categorical issues that imply a systematic relationship between obesity and psycho-pathological outcomes. However, certain research contradicts this notion and asserts that specific obese individuals face a higher risk of psychiatric disorders, particularly depression, which may not entirely return to normal after weight reduction [24]. These disparities could arise from differing genetic backgrounds and the varied impact of diets. Ultimately, the findings reinforce the potential role of lifestyle adjustments in fostering healthier living for OW participants, possibly reducing associated psychological risks.

The research also uncovered correlations between phyla abundance and eating patterns, particularly among post-intervening OW/OB participants who regularly or occasionally consumed vegetables and fruits. Interestingly, a higher abundance of Firmicutes was also found among those who did not eat out and participants engaging in regular physical activities. Concerning cytokine parameters, elevated TNF-alpha levels corresponded with a significant increase in Bacteroidetes abundance among OW/OB participants. Likewise, a positive relationship was seen between the relative abundance of Firmicutes and increased TNF-α levels correlating with obesity in children [25]. Furthermore, higher TNF-alpha and IL-6 levels were linked with greater Firmicutes abundance in post-intervening OW/OB individuals compared to healthy peers.

Mental health characteristics are linked to gut microbiota compositions, as supported by numerous global studies [12,26,27]. Increased Bacteroidetes prevalence was noted in healthy weight and post-intervening OW/OB individuals exhibiting specific traits such as atypical non-categorical negative suicidality, frequent internalization and attention to problems, and a standard overall mental health score. The Bacteroide phyla plays a role in regulating psychological well-being. In alignment with these findings, it has been demonstrated that a Bacteroides-dominant gut microbiome in late infancy correlates with improved neurodevelopment within a Canadian Healthy Infant Longitudinal Development Cohort [28]. The results also indicate a discernible connection between inflammatory and psychological factors with different phyla in OW/OB and healthy participants. Specifically, a higher prevalence of Firmicutes was detected among post-intervening OW/OB individuals relative to healthy ones across various categories, such as regular physical activity, fruit and vegetable consumption, and avoiding eating out. Conversely, a higher abundance of Bacteroidetes was found among OW/OB individuals, demonstrating elevated TNF-alpha levels compared to post-intervening OW/OB participants. 

Research analyzing functional pathways demonstrated that most KOs were associated with metabolic pathways. Significant disparities between OW/OB and healthy-weight individuals were revealed when broken down into subcategories. Although no KOs were statistically significant after accounting for multiple comparisons, it was apparent that various metabolic processes were more prevalent in pre-intervening OW/OB individuals than in their healthy-weight counterparts. These processes encompass fructose and mannose metabolism, glycolysis, gluconeogenesis, nucleotide sugar biosynthesis, starch, and sucrose metabolism.

Examining post-intervention OW/OB and healthy participants also highlighted substantial differences in functional profiles related to carbohydrate metabolism. In post-intervention OW/OB subjects compared to those of healthy weight, 433 KOs exhibited higher abundance. Some were affiliated with metabolic pathways, biosynthesis, and metabolism—potentially elucidating a mechanism through which lifestyle modification impacts obesity regulation. Several researchers have already reported on gut microbiomes altering diverse metabolic pathways [29,30]. These energy and glucose metabolism pathways play a role in regulating various psychological parameters [31].

A comparison of the functional profiles between post-intervention and pre-intervention OW/OB individuals revealed statistically significant differences prior to adjusting for multiple testing. Nonetheless, no KOs remained significant post-correction. The differentially abundant KOs pertained to two-component systems, co-factor biosynthesis or amino acid production, or other non-carbohydrate-related metabolic pathways. Abundant bacteria such as Firmicutes and Bacteroidetes are known to participate in the metabolism of various amino acids and co-factors, influencing brain psychology and inflammatory parameters [32]. The study showed some effects of obesity on psychological and mental health parameters; however, the major limitations of the study were the low number of participants and the short duration of the study. Future studies would be enhanced by recruiting more participants and having longer interventions and follow-up periods. 

## 5. Conclusions

In conclusion, the study emphasizes the benefits of lifestyle modifications in improving mental wellness and reducing inflammation in overweight adolescents. It reveals the relationship between gut microbiota and psychological factors and demonstrates how lifestyle changes impact the microbiomes of overweight youth. Ultimately, this knowledge could lead to innovative obesity treatments targeting specific microbial shifts and promoting personalized lifestyle-based interventions for obesity management.

## Figures and Tables

**Table 1:** Social characteristics and anthropometric measurements of healthy and OW/OB adolescents; 22 total participants consisting of 10 boys and 12 girls.

Parameters (M ± SD or *n*)		Healthy (*n* = 16)	OW/OB (*n* = 22)
Gender	Male (*n* = 17)	7	10
	Female (*n* = 21)	9	12
Age (years)	8–12 (*n* = 18)	9.5 ± 1.4	10.1 ± 1.36
	13–18 (*n* = 16)	16.1 ± 0.98	14.77 ± 1.74
Hight (cm)		153.8 ± 17.8	164.3 ± 11.8
Weight (kg)		47.4 ± 16.7	63.5 ± 42.4
Race	African American (*n* = 3)	1	2
	Hispanic (*n* = 9)	3	6
	Caucasian (*n* = 18)	5	0
	Mix-race (*n* = 6)	2	4
Body Mass Index (BMI) (kg/m^2^)		19.4 ± 3.0	31.4 ± 5.2
BMI-z score		0.23 ± 0.57	2.22 ± 0.34
Waist-to-hip ratio		0.67 ± 0.21	0.72 ± 0.41

*n*, number of the participants. BMI z-scores are used to evaluate relative weight adjusted for child age and sex. BMI and BMI z-score have been calculated using clinical calculator from Merck Manual. (https://www.merckmanuals.com/professional/pages-with-widgets/clinical-calculators?mode=list, accessed on 26 September 2023).

**Table 2:** Association of relative abundance of dominate gut microbiome phylum with lifestyle modification, urinary inflammation marker, and psychological parameters among the different children groups.

Phylum	Characteristics	Category	Weight Attribute Comparison	Median Difference (%)	Adjusted *p*-Value *
Lifestyle characteristics					
Firmicutes	Physical activities	Regular	Healthy- OW/OB	−6.25	0.037
Firmicutes	Physical activities	Regular	OW/OB vs. OW/OB post-intervention	−22.55	0.022
Firmicutes	Physical activities	Regular	Healthy vs. OW/OB post-intervention	−28.79	0.000699
Firmicutes	Vegetable consumption	Regular	Healthy vs. OW/OB post-intervention	−28.91	0.013
Firmicutes	Vegetable consumption	Sometimes	Healthy vs. OW/OB post-intervention	−14.88	0.002
Firmicutes	Fruit consumption	Sometimes	Healthy vs. OW/OB post-intervention	−13.57	0.002
Firmicutes	Meals outside	No	OW/OB vs. OW/OB post-intervention	−8.97	0.031
Firmicutes	Meals outside	No	Healthy vs. OW/OB post-intervention	−13.09	0.008
Firmicutes	Meals outside	Sometimes	Healthy vs. OW/OB post-intervention	−33.97	0.008
Cytokine parameters					
Bacteroidetes	TNF-alpha level	Higher	OW/OB vs. OW/OB post-intervention	20.57	0.009
Firmicutes	TNF-alpha level	Higher	Healthy vs. OW/OB post-intervention	−23.58	0.001
Firmicutes	TNF-alpha level	Higher	OW/OB vs. OW/OB post-intervention	−15.27	0.01
Firmicutes	IL-6 level	Normal	OW/OB vs. OW/OB post-intervention	−11.54	0.017
Firmicutes	IL-6 level	Normal	Healthy vs. OW/OB post-intervention	−15.07	0.000495
Bacteroidetes	ET1 level	Higher	Healthy vs. OW/OB post-intervention	33.85	0.019
Firmicutes	ET1 level	Higher	Healthy vs. OW/OB post-intervention	−38.75	0.005
Firmicutes	ET1 level	Normal	Healthy vs. OW/OB post-intervention	−15.54	0.004
Mental health attributes					
Bacteroidetes	Internalizing problem	Frequent	Healthy vs. OW/OB post-intervention	18.38	0.036
Bacteroidetes	Internalizing problem	Frequent	OW/OB vs. OW/OB post-intervention	12.32	0.036
Firmicutes	Internalizing problem	Frequent	OW/OB vs. OW/OB post-intervention	−10.76	0.015
Firmicutes	Internalizing problem	Frequent	Healthy vs. OW/OB post-intervention	−13.01	0.00075
Bacteroidetes	Attention problem	Frequent	Healthy vs. OW/OB post-intervention	28.23	0.026
Bacteroidetes	Attention problem	Frequent	OW/OB vs. OW/OB post-intervention	15.01	0.026
Firmicutes	Attention problem	Frequent	OW/OB vs. OW/OB post-intervention	−10.95	0.047
Firmicutes	Attention problem	Frequent	Healthy vs. OW/OB post-intervention	−16.85	0.000274
Firmicutes	Externalizing problem	Frequent	OW/OB vs. OW/OB post-intervention	−12.15	0.036
Firmicutes	Externalizing problem	Frequent	Healthy vs. OW/OB post-intervention	−21.04	0.007
Bacteroidetes	Suicidality	Negative	Healthy vs. OW/OB post-intervention	22.79	0.015
Bacteroidetes	Suicidality	Negative	OW/OB vs. OW/OB post-intervention	12.42	0.041
Firmicutes	Suicidality	Negative	OW/OB vs. OW/OB post-intervention	−12.13	0.009
Firmicutes	Suicidality	Negative	Healthy vs. OW/OB post-intervention	−15.54	0.0000022
Bacteroidetes	Non-categorical	Abnormal	Healthy vs. OW/OB post-intervention	20.8	0.013
Bacteroidetes	Non-categorical	Abnormal	OW/OB vs. OW/OB post-intervention	14.99	0.033
Firmicutes	Non-categorical	Abnormal	OW/OB vs. OW/OB post-intervention	−10.74	0.013
Firmicutes	Non-categorical	Abnormal	Healthy vs. OW/OB post-intervention	−14.04	0.0000241
Bacteroidetes	Total score	Normal	Healthy vs. OW/OB post-intervention	22.68	0.013
Bacteroidetes	Total score	Normal	OW/OBvs OW/OB post-intervention	12.56	0.04
Firmicutes	Total score	Normal	OW/OB vs. OW/OB post-intervention	−13.51	0.007
Firmicutes	Total score	Normal	Healthy vs. OW/OB post-intervention	−17.04	0.00000155

* *p*-value was adjusted for multiple comparisons using Bonferroni correction.

## Data Availability

The clinical data analyzed in this study are not publicly available because individual privacy may be compromised. However, the study protocol and statistical analysis plan will be shared upon request. In addition, 16s bacterial seducing data will be submitted to NCBI and publicly shared upon publication of the manuscript.

## Supplementary Materials

The following supporting information can be downloaded at: https://www.mdpi.com/article/10.3390/nu15194243/s1, Figure S1: Relative abundance of gut microbiota phyla between healthy weight, pre-intervening overweight/obese (OW/OB), and post-intervention OW/OB participants. Figure S2: Relative abundance of all the gut microbiota genera between healthy weight, pre-intervening OW/OB, and post-intervention OW/OB participants. Figure S3: Relative abundance of all the gut microbiota genera between healthy weight, pre-intervening OW/OB, and post-intervention OW/OB participants within Actinobacteria phyla. Figure S4: Nonmetric multidimensional scaling (NMDS) plot of the Bray–Curtis dissimilarity for microbial community’s differences between the healthy weight, pre-intervention OW/OB, and post-intervention OW/OB participants. Each dot represents the bacterial community of each participant and is colored by weight attributes. Ellipses represent standard error around the centroid of each weight attribute and are colored by weight attribute. Bacterial community composition between the healthy, pre-intervention OW/OB, and post-intervention OW/OB participants differ and are distinct (PERMANOVA, *p* < 0.0001). Figure S5: (A) Box plot showing the differential relative abundance of carbohydrate metabolism-related KOs between post-intervention OW/OB (red color) and healthy weight participants (other/blue color). (B) Box plots showing the differential relative abundance of KOs unrelated to carbohydrate metabolism and No carbohydrate metabolism KOs were statistically significant in post-intervention OW/OB participants.
