# Supplementary material for: Change in Urinary Inflammatory Biomarkers and Psychological Health with Gut Microbiome Modulation after Six Months of a Lifestyle Modification Program in Children
Source: Nutrients. 2023 Oct 1;15(19):4243. doi: 10.3390/nu15194243 (PMC10574711; doi:10.3390/nu15194243)
Supplement: Supplementary file 1 [file nutrients-15-04243-s001.zip › Supplementary S1.pdf]

Section A: Details of Lifestyle Modification Program

Session 1	The first session was dedicated to introducing and assessing knowledge of measurements, which could promote weight loss behavior. The assessment was done toward knowledge about nutrition and physical activity. Parents were educated on becoming role models to create a healthy home environment and encourage family engagement and decision-making to stay healthy and fight obesity. An overview was given on how to help their children to eat well and move more. The discussion also focused on the long-term consequences of obesity for overall health.
Session 2	The session focused on healthy nutrition education and how to implement it for younger and older children. Drastic dietary changes were not encouraged, but recommendations such as buying less fatty milk were given. The families were recommended to place a bowl of washed fruit on a table and consider it an easily available snack. The recommended food should be lower in calories, fat, and sugar. Physical activity as an additional energy loss was discussed.
Session 3	The session was focused on the energy balance. The definition of energy balance was done, and portion size and serving size were effective ways to control weight. Participants were also asked about their ability to implement previous session recommendations.
Session 4	The session discussed in depth how to cook with low fat methods such as baking, broiling, boiling vs. frying' and how to choose low fat or fat free dairy products, salad dressing, mayonnaise, etc. During this session, we also introduced the concept of reducing screen time.

Session 5	The session mostly focused on physical activity and making it fun. The discussion was arranged around adding more physical activity into the family's daily lives and the challenges a family may have. Role of physical activity in energy balance and needs "energy out." Discussed physical activity and worked out on emotional status and how to incorporate short periods of physical activities (15-30 minutes) in daily routine. During this session a decrease in screen time was also discussed in more detail. Evaluate how much time a family spends in front of a screen (T.V., DVDs, non-school or work-related computer use)—discuss how to limit screen time to no more than 2 hours for non-work related activity.
Session 6	The focus was on maintaining enquired knowledge, maintaining a healthy weight, and making it lifelong habits. The family was educated that weight maintenance is a "marathon" with strong motivation. Finding a network and support groups who were into healthy lifestyles was encouraged.

Section B: Details bioinformatic analysis bacterial 16S genome sequencing data and their association with clinical psychological parameters

The raw data was downloaded was processed using the BLC2FASTQ software developed by Illumina (https://illumina.com). This software converts the raw data from the NGS Microbiome experiment into the read-format.

1. The Array-Star software, part of the Next Generation Sequence (NGS) genomics analysis suite of softwares, DNAStar (www.dnastar.com) was used to map 184,355 reads to the 16S genes of 3.8 million individual bacterial species. The 16S bacterial gene information was accessed from the Ribosomal Data Project (RDP rdp.cme.msu.edu) resource housed at Michigan

State University. The microbiome mapping feature in ArrayStar was chosen. The reads from one or more samples mapped to 1,410 independent bacterial species.   The raw reads were then normalized for every identified bacterial species down each sample. The normalized values of the reads were then additionally processed to identify the bacteria to which the reads were mapped.

2 A PERL (Practical Extraction and Report Language) program was written to extract taxonomic information up to the level of an OTU for every bacterial species identified from the experiments. The taxonomies for bacterial species (the 16S gene) in RDP are varied. However, the PERL program followed by manual oversight revealed that the OTU for each species in the samples was at the taxonomic level of "Family."

3. After that, OUT data were imported and managed using Microsoft Excel 365 for Windows (2021, Microsoft Corp., Redmond, WA, USA) and then analyzed using R software computing environment using different R packages (v4.2.2) for following analysis.

4. Relative abundance and alpha diversity indices: Relative abundances for the total reads for the identified operational taxonomic units (OTUs) of the gut microbiota of the study participants based on weight attributes were explored and presented using bar plots and boxplots. We assessed Alpha diversity in the gut microbiota of the study participants using Chao's richness index as a measure of the uniqueness of OTUs and Shannon's Diversity Index as a measure of microbial richness and evenness. We statistically explored any possible relationship and differences in relative abundance of dominant gut microbiota, and alpha diversity indices between the weight attributes (healthy, OW/OB, and OW/OB post-intervention groups), lifestyle characteristics, cytokine parameters, and mental health attributes of the study participants using

Mann-Whitney (MW) non-parametric test. Adjustment for multiple comparison in all comparison be-tween different attributes was achieved using Bonferroni correction.

5. Beta diversity analysis: Beta-diversity analysis of the gut microbiota among the study participants was evaluated using the Bray–Curtis dissimilarity index. Beta diversity between the weight attribute groups was further visualized using nonmetric multi-dimensional scaling (NMDS) ordination plot. An initial beta-dispersion analysis was done to ensure equal dispersion assumption between the groups. Permutational multivariate analysis of variance (PERMANOVA) was further used to statistically test the differences in distance and association of beta diversity between the weight attribute groups, lifestyle characteristics, cytokine parameters, and mental health attributes of the study participants. Pairwise comparisons between predicting factors were assessed and P-values were adjusted for multiple comparisons using Bonferroni correction.

6. Differential abundance of gut microbiota: We used similarity percentages analysis (SIMPER) to investigate the specific OTUs driving microbial community differences between the weight attribute groups. Specific OTUs that accounted for >1% of the microbial community difference were investigated between the weight attribute groups using Kruskal-Wallis (KW) non-parametric test. Pairwise comparisons between the weight attribute groups were assessed and P-values were adjusted for multiple comparisons using Benjamini–Hochberg method of False Discovery Rate to confirm differential abundance.

7. Functional profile and differential analysis: Functional capacity and characteristics of the gut microbiota of the study participants was carried out using NAMCO Micro-biome Explorer

v1.1 (Dietrich et al., 2022). The built-in PICRUSt2 pipeline and option (Langille et al., 2013) of NAMCO Microbiome Explorer was used to predict the func-tional Kyoto Encyclopedia of Genes and Genomes (KEGG) orthology profile for each sample from the relative abundance of OTUs. The relative abundance of average copy numbers of the KEGG orthologues (KO) profiles was normalized and aggregated into functional pathways and categories based on KEGG annotations in order to under-stand their functions. Differential abundance of KO profiles was statistically com-pared between the weight attribute groups using MW non-parametric test adjusting for multiple comparison using Benjamini–Hochberg method of False Discovery Rate. 8.
