# Supplementary material for: Change in Urinary Inflammatory Biomarkers and Psychological Health with Gut Microbiome Modulation after Six Months of a Lifestyle Modification Program in Children
Source: Nutrients. 2023 Oct 1;15(19):4243. doi: 10.3390/nu15194243 (PMC10574711; doi:10.3390/nu15194243)
Supplement: Supplementary file 1 [file nutrients-15-04243-s001.zip › Table S1.pdf]

Table S1. Anthropometric and Metabolic Profiles of OW/OB Adolescents. Calculated averages of different parameters of each OW/OB patient.

Parameters	Males (n=10) (Average)	Females (n=12) (Average)
**Systolic Blood Pressure Percentile (%)**	79.4 ± 23.1	62.5 ± 32.2
**Diastolic Blood Pressure Percentile (%)**	70.3 ± 27.0	69.9 ± 28.7
**Total Cholesterol**	159.8 ± 30.76	159.7 ± 24.65
**Triglycerides**	146.5 ± 91.87	115.9 ± 47.95
**LDL**	88.4 ± 33.4	95.1 ± 24.9
**HDL**	50.0 ± 24.9	44.8 ± 10.4
**HbA1c**	5.4 ± 0.43	5.4 ± 0.30
**Body Mass Index (BMI) z-scores**	2.35 ± 0.280	2.18 ± 0.353
**Waist-to-hip Ratio**	1.1 ± 0.32	0.84 ± 0.077
