# Supplementary figures and images for: Change in Urinary Inflammatory Biomarkers and Psychological Health with Gut Microbiome Modulation after Six Months of a Lifestyle Modification Program in Children
Source: Nutrients. 2023 Oct 1;15(19):4243. doi: 10.3390/nu15194243 (PMC10574711; doi:10.3390/nu15194243)

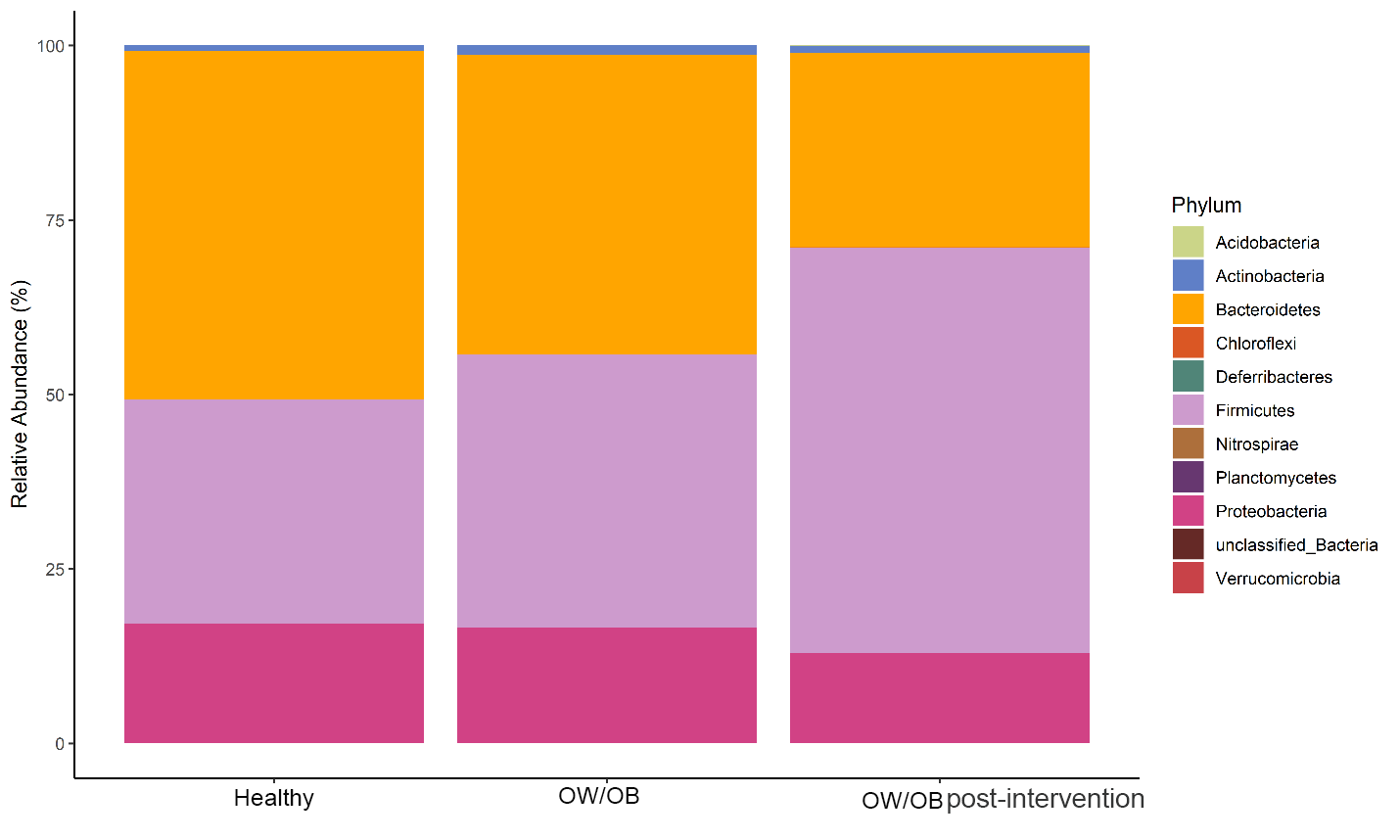

Supplement: Supplementary file 1 [file nutrients-15-04243-s001.zip › Fig S1.tif]

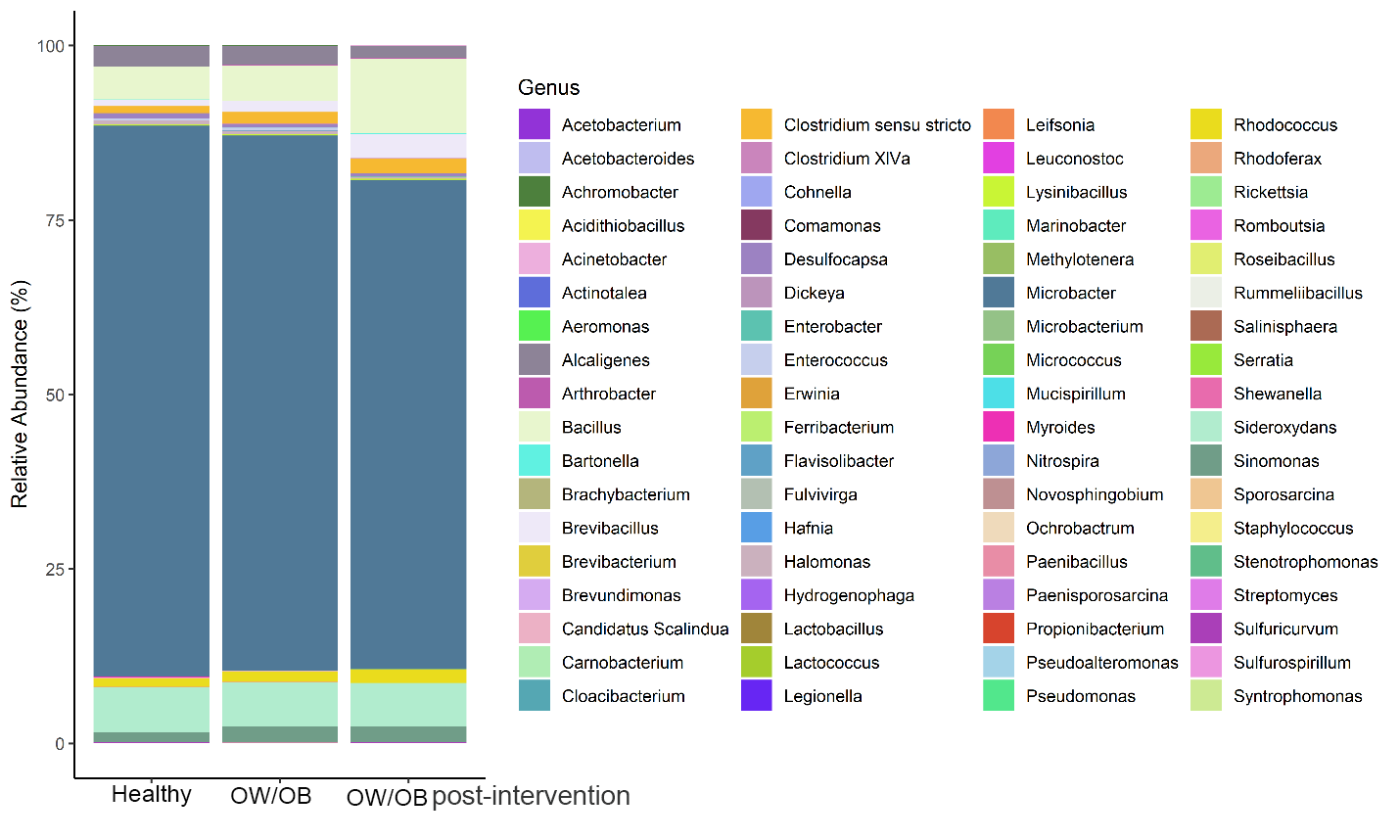

Supplement: Supplementary file 1 [file nutrients-15-04243-s001.zip › Fig S2.tif]

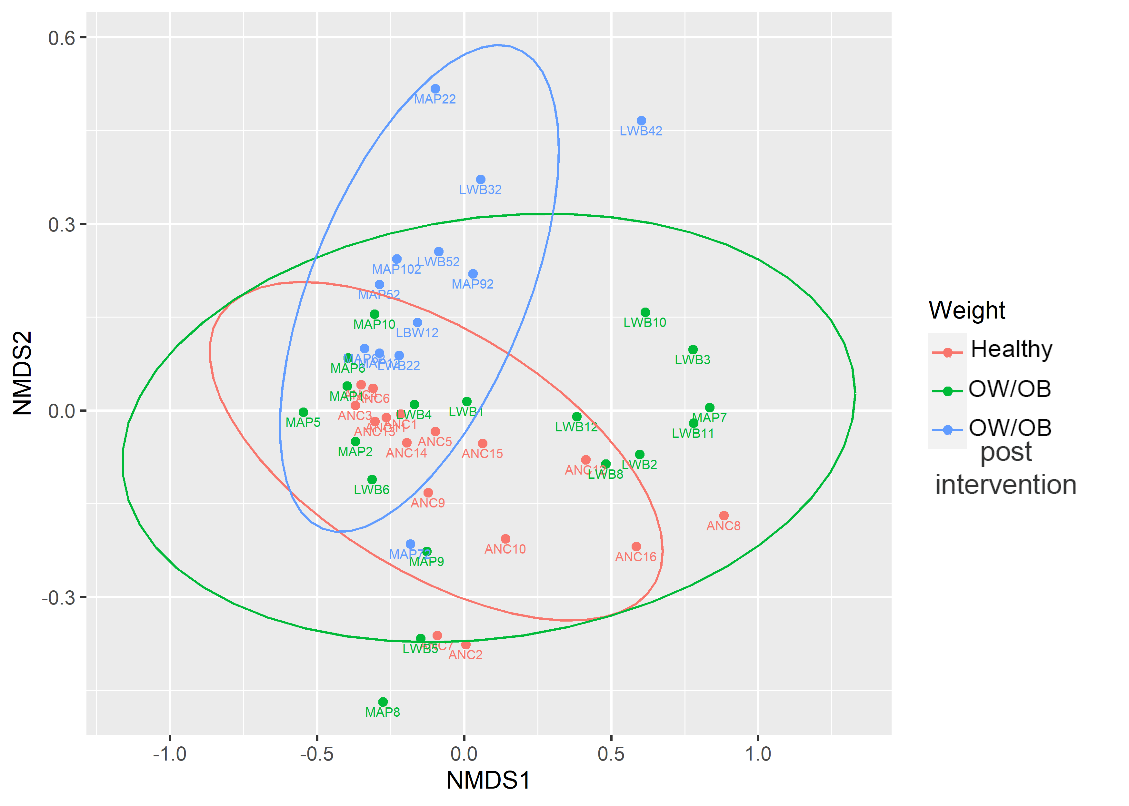

Supplement: Supplementary file 1 [file nutrients-15-04243-s001.zip › Fig S3.tif]

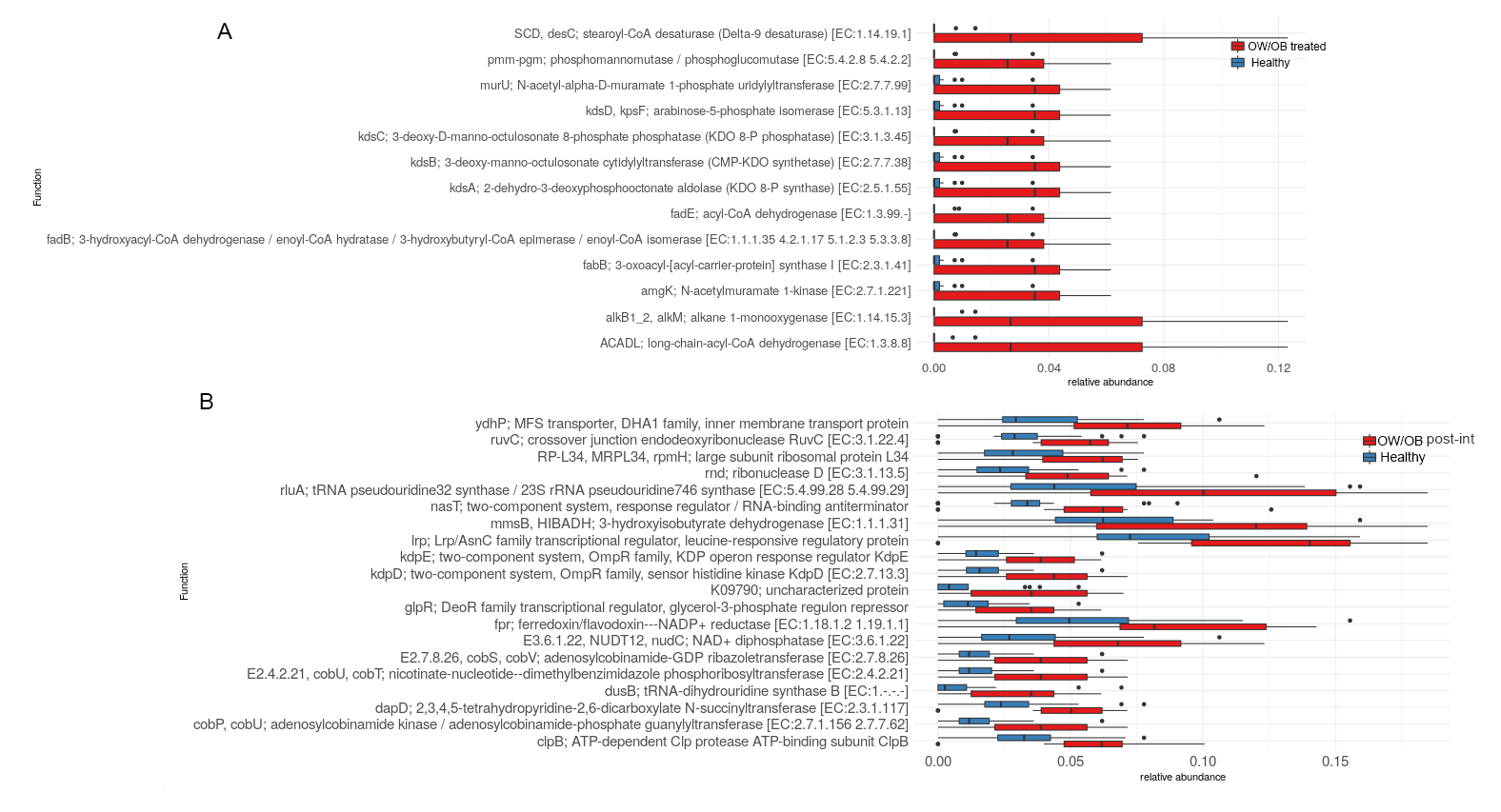

Supplement: Supplementary file 1 [file nutrients-15-04243-s001.zip › Fig S4.tif]

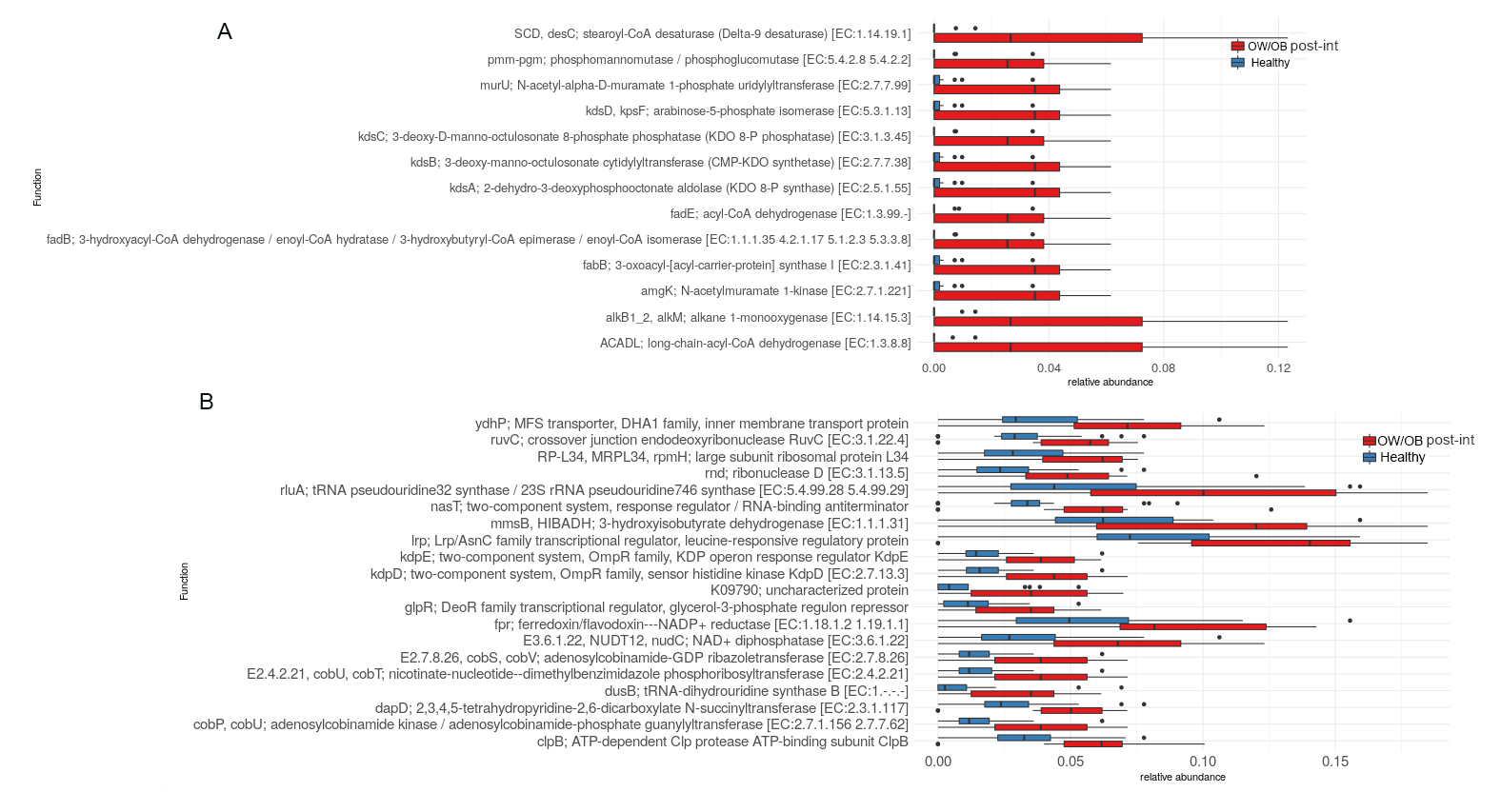

Supplement: Supplementary file 1 [file nutrients-15-04243-s001.zip › Fig S4a.tif]

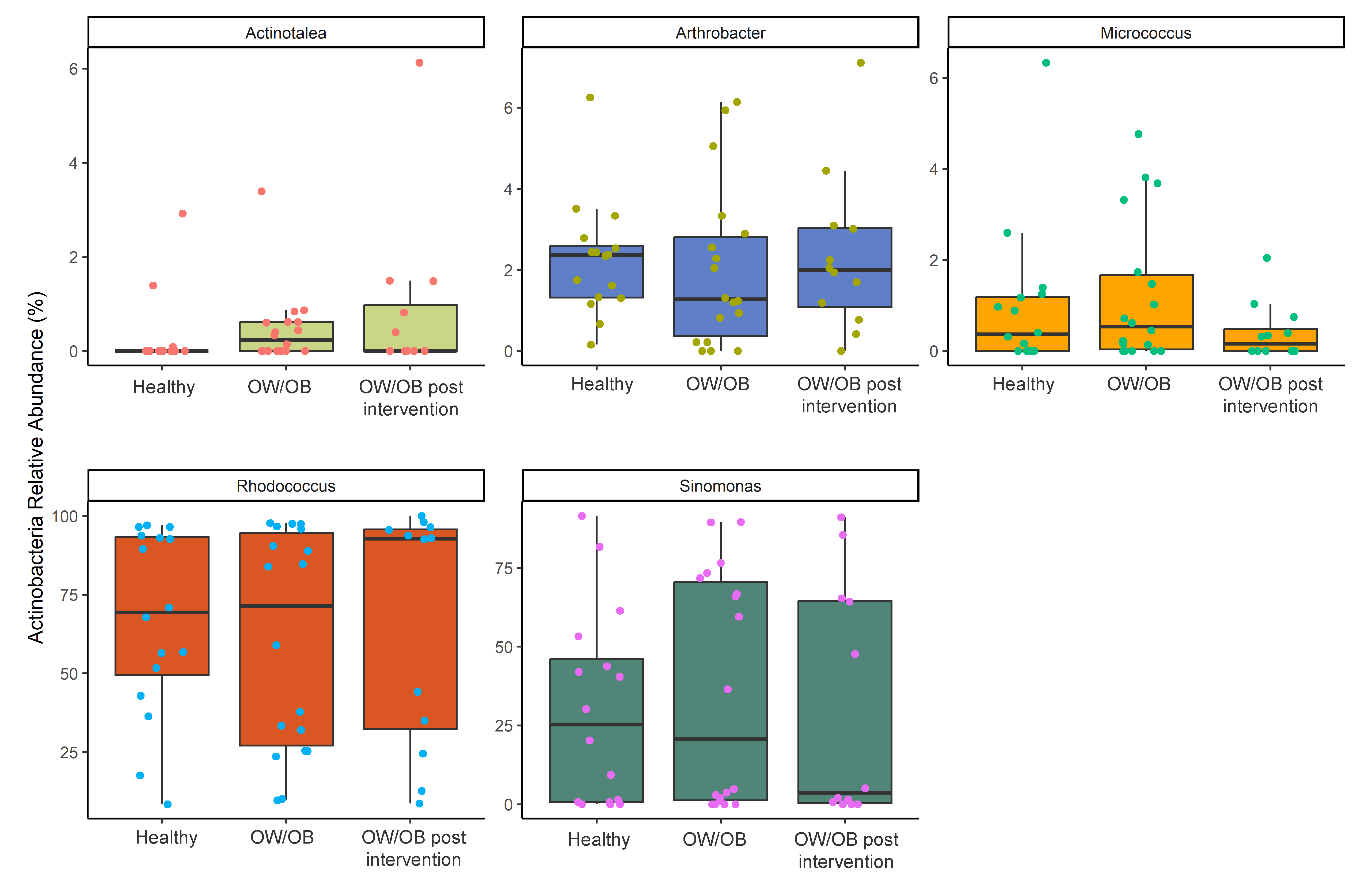

Supplement: Supplementary file 1 [file nutrients-15-04243-s001.zip › Fig S5.tif]
